# Who drops out and when? Predictors of non-response and loss to follow-up in a longitudinal cohort study among STI clinic visitors

**DOI:** 10.1371/journal.pone.0218658

**Published:** 2019-06-19

**Authors:** Daphne A. van Wees, Chantal den Daas, Mirjam E. E. Kretzschmar, Janneke C. M. Heijne

**Affiliations:** 1 Centre for Infectious Disease Control, National Institute for Public Health and the Environment, Bilthoven, The Netherlands; 2 Department of Interdisciplinary Social Science, Faculty of Social and Behavioral Sciences, Utrecht University, Utrecht, The Netherlands; 3 Julius Centre for Health Sciences and Primary Care, University Medical Centre Utrecht, Utrecht University, Utrecht, The Netherlands; David Geffen School of Medicine at UCLA, UNITED STATES

## Abstract

**Introduction:**

Response rates in health research are declining, and low response rates could result in biased outcomes when population characteristics of participants systematically differ from the non-respondents. Few studies have examined key factors of non-response beyond demographic characteristics, such as behavioral and psychological factors. The aim of the current study was to identify predictors of non-response and loss to follow-up in a longitudinal sexual health study.

**Materials and methods:**

A longitudinal cohort study (iMPaCT) was conducted from November 2016 to July 2018 among heterosexual STI clinic visitors aged 18–24 years. At four different time points in one year, data was collected on sexual behavior, psychological determinants and chlamydia infections. The national STI surveillance database provided data on demographic, behavioral and sexual health-related characteristics for non-respondents. Predictors of non-response at baseline and of loss to follow-up were identified using multivariable logistic regression analyses.

**Results:**

In total, 13,658 STI clinic visitors were eligible to participate, of which 1,063 (8%) participated. Male gender, low/medium education level, young age (≤ 20 years) and having a non-Dutch migration background were significant predictors of non-response at baseline. Furthermore, non-respondents at baseline were more likely to report STI-related symptoms, to have been notified by a partner, to have had condomless sex, and to have had ≤ 2 partners in the past six months, compared to participants. Psychological predictors of loss to follow-up differed between STI clinic regions, but low perceived importance of health at baseline was associated with loss to follow-up in all regions. The baseline chlamydia positivity rate was significantly higher in the non-respondents (17%) compared to the participants (14%), but was not a predictor of loss to follow-up.

**Discussion:**

Targeted recruitment aimed at underrepresented groups in the population based on demographic, behavioral and psychological characteristics, might be necessary to decrease loss to follow-up, and to prevent non-response bias in health research.

## Introduction

Response rates in health research have been declining over the past decades [1, 2]. This decline is concerning, especially because low response rates can lead to systematic differences in population characteristics between participants and non-respondents [3–5]. These differences in characteristics, hereafter referred to as non-response bias, could result in biased study outcomes [1, 2, 6, 7]. Strategies, such as data weighting techniques, can be used to reduce non-response bias, but the extent to which these strategies minimize bias is dependent on the availability of data on all potential factors that might be associated with non-response [8]. This highlights the importance of gaining insight into key factors of non-response.

Most studies exploring key factors of non-response examined differences in demographic characteristics between participants and non-respondents. These studies found that females, highly educated, or older individuals (> 24 years), are usually more likely to participate in health research than males, lower educated, or younger individuals (≤ 24 years) [3, 5, 8, 9]. Fewer studies also compared behavioral characteristics of participants and non-respondents. In sexual health-related research, these studies found that low-risk individuals, in terms of lower number of sexual partners or less often previously diagnosed with a sexually transmitted infection (STI), were less likely to participate than high-risk individuals [5, 10, 11]. One study explored psychological characteristics in sexual health research context and found that non-respondents had less reward dependent and more harm-avoidant personalities than participants [12]. These findings suggest that identifying key factors of non-response should not be limited to only demographic characteristics.

In addition to low response rates during recruitment, loss to follow-up in longitudinal cohort studies could also lead to non-response bias when characteristics and health outcomes of participants lost to follow-up are different from the participants who are retained in the study [13–15]. Previous research indicated that certain demographic characteristics related to non-response at recruitment, such as lower education or low socioeconomic status, were also associated with loss to follow-up in longitudinal cohort studies [14, 15]. Although some empirical studies identified general psychological drivers behind participation in longitudinal studies [16, 17], such as positive attitudes towards participating in surveys, few such studies were conducted in sexual health-related research. One study in sexual health context, found that patients with poor knowledge of STI and a higher level of perceived STI-related stigma were more likely to be lost to follow-up from STI care [18]. However, this study was conducted in a low-resource setting and only included STI diagnosed individuals.

The objectives of this study were to identify demographic, sexual health-related, and behavioral predictors of non-response during recruitment, and to identify demographic, sexual health-related, behavioral, and psychological predictors of loss to follow-up. Data from a longitudinal cohort study in the Netherlands called 'Mathematical models incorporating Psychological determinants: control of Chlamydia Transmission' (iMPaCT) offered a unique opportunity to analyze comprehensive information of both participants and non-participants during recruitment and loss to follow-up.

## Materials and methods

### Setting

A detailed description of the study design can be found in the iMPaCT study protocol [19]. In short, the iMPaCT study explored the link between sexual behavior, psychological determinants, and chlamydia infections over a period of one year, using online questionnaires and information routinely registered by STI clinics. All heterosexual males and females, and females who have sex with both males and females, aged 18 to 24 years, making an appointment at the STI clinics of the public health services in Amsterdam, Kennemerland, Hollands Noorden, and Twente in the Netherlands from November 2016 to June 2017 were eligible to participate in the iMPaCT study. Two different recruitment strategies were used. At the STI clinics in Amsterdam, Kennemerland, and Hollands-Noorden, individuals were invited to participate in the iMPaCT study after finishing the online intake assessment for an STI test. After agreeing to participate and signing the online informed consent form, they were automatically redirected to the online questionnaire assessing psychological and behavioral determinants (see S1 and S2 Files for original and English translation of the questionnaire, and the invitation). At the STI clinic in Twente, it was only possible to make an appointment by telephone, and individuals were informed about the iMPaCT study at the end of the intake assessment. If they agreed to participate, they received an e-mail with the link to the informed consent and the online questionnaire. Individuals who agreed to participate, provided informed consent and started the baseline questionnaire will hereafter be referred to as *participants*, and all other eligible STI clinic visitors will be referred to as *non-respondents*.

Participants were enrolled for one year, and online questionnaires were administered at four different time points: baseline, three-week follow-up, six-month follow-up, and one-year follow-up. Furthermore, participants were tested for chlamydia using nucleic acid amplification tests (NAAT) at enrolment at the STI clinic and through a self-sampling kit sent to a laboratory at six-month follow-up. Participants were invited for all follow-up data collection moments, if they completed the baseline questionnaire and provided a valid email address. Participants could be temporarily lost to follow-up (i.e., completed questionnaire at six-month and one-year follow-up, but did not respond to the three-week follow-up questionnaire) or permanently (i.e., completed questionnaire at three-week follow-up, but did not respond to subsequent questionnaires).

Several passive recruitment strategies were used to increase response rates and retention in the iMPaCT study. First, the STI clinic visitors were informed of receiving a free of charge home-based test kit for chlamydia and gonorrhea after completing the questionnaire at six-month follow-up, and a monetary incentive after completing the questionnaire at one-year follow-up. Second, during follow-up the participants received two reminders per questionnaire by e-mail: one week and two weeks after the invitation for each follow-up questionnaire. The iMPaCT study was approved by the Medical Ethical Committee of the University Medical Centre Utrecht, the Netherlands (NL57481.094.16/METC18-363/D).

### Data non-response at baseline

The national STI surveillance database provided consultation data of all eligible STI clinic visitors during the recruitment period (November 2016—June 2017) in the four participating STI clinics. Participants were distinguished from the non-respondents in the consultation data using a unique iMPaCT study identification number. For individuals who visited the STI clinic more than once in the recruitment period, only the first consultation was included in the analyses, or, in case they agreed to participate in the iMPaCT study, the consultation linked to the iMPaCT questionnaire was included.

Demographic information obtained from the national STI surveillance database included age, gender (female/male), education level defined as highest attained degree or education currently enrolled in (low/medium: *no education*, *primary education only*, *lower general secondary education and vocational education*, high: *all other education levels*), STI clinic region (Amsterdam/Non-Amsterdam: *Hollands Noorden/Kennemerland/Twente*), and migration background (ethnic Dutch/non-Dutch). Migration background was based on the birth country of the participant and both parents consistent to the definitions used by Statistics Netherlands [20] and was categorized into two groups: Dutch (participant and both parents are born in the Netherlands) and non-Dutch (first-generation and second generation migrants from all other countries).Sexual health-related information included the STI test results at baseline (positive at any anatomic location/negative), type of STI test at baseline (regular consultation/self-sampling test kit), as well as STI-test in the past year, prior chlamydia/gonorrhea/syphilis diagnosis in the past year, and STI-related symptoms (all yes/no). Finally, the STI surveillance databases contained information on sexual behavior, including number of partners in the past six months, received partner notification (yes/no), and condom use most recent sex act (yes/no).

### Data loss to follow-up

For participants, additional data on behavioral and psychological data was available from the baseline online questionnaire. The development of the questionnaire has been described in detail in the iMPaCT study protocol [19]. Behavioral data included the number of sexual partners in the past six months, and age at sexual debut. Psychological data included perceived importance of (sexual) health or “*health goals”*, attitudes regarding prevention of chlamydia, intentions towards condom use and STI testing in the future, anticipated stigma, shame and anxiety with regard to chlamydia diagnosis, self-efficacy regarding condom use, expected social support after chlamydia diagnosis, subjective and social norms regarding condom use and STI testing, self-esteem, impulsiveness, risk perception, and knowledge regarding sexual health, prevention of chlamydia and consequences of chlamydia diagnosis.

The psychological characteristics were assessed on 5-point Likert scales, except for risk perception and knowledge. The 5-point Likert scales ranged from 1 (i.e., low level of the determinant) to 5 (i.e., high level of the determinant), and a mean score was calculated for all psychological scales. Risk perception was assessed on a scale from 0% to 100%. Risk perception for chlamydia *own risk* was defined as the mean of the participants’ estimate of their own risk in the coming year and in their lifetime, and risk perception for chlamydia *peers’ risk* was defined as the mean of the participants’ estimate of the risk of their peers in the coming year and in their lifetime. Knowledge of sexual health in terms of prevention of chlamydia and consequences of chlamydia diagnosis, was assessed by six *true/false/I don’t know* items, and was defined as the sum score of six items based on the number of correct answers (zero to six). Subjective and social norms, and social support were combined into one variable reflecting social environment. The score of each psychological determinant was divided in two categories at the median: low/median = lower than median, high = equal to or higher than median.

### Statistical analyses

To identify predictors for non-response at baseline, univariable and multivariable logistic regression analyses were performed using data from the national STI surveillance database on participants and non-respondents. All demographic, sexual health-related, and behavioral baseline characteristics were included in the univariable and multivariable analyses. Chlamydia positivity rates at baseline were compared between participants and non-respondents using a chi-squared test.

Predictors for loss to follow-up were also identified with univariable and multivariable logistic regression analyses, using data collected at baseline. The response to each follow-up moment was analyzed separately, as participants could be temporarily lost to follow-up. First, baseline characteristics were compared between participants who completed the questionnaire at three-week follow-up and participants who did not respond to the questionnaire invitation at three-week follow-up. Second, baseline characteristics were compared between participants who completed the questionnaire at six-month follow-up and participants who did not respond to the questionnaire invitation at six-month follow-up. Last, baseline characteristics were compared between participants who completed the questionnaire at one-year follow-up and participants who did not respond to the questionnaire invitation at one-year follow-up. In the univariable analyses, the baseline chlamydia test results, and the behavioral and psychological variables from the baseline questionnaire, were included in addition to the demographic, sexual health-related and behavioral variables from the national STI surveillance database. As the number of potential variables for the multivariable model was relatively high in relation to the sample size, the baseline variables included in the multivariable analyses were pre-selected in the univariable analyses using a p-value criterion of 0.1. Variables were excluded from the multivariable model if the number of observations per outcome category was too small in relation to the number of predictors [21]. If a variable was associated with loss to follow-up at either the three-week, six-month and/or one-year follow-up in the univariable analyses, it was included in all multivariable models.

All multivariable models (non-response and loss to follow-up) were constructed using a backward elimination procedure, based on the lowest Akaike information criterion (AIC) score. Interaction terms were added to the multivariable model, and if statistically significant, stratified analyses were shown. Missing values were included as a separate category if more than 5% of the observations were missing. Multicollinearity was evaluated using the Variance Inflation Factor (VIF) (VIF values above 5 indicating multicollinearity) [22], and variables that were highly correlated with other predictors were removed from the multivariable model. Goodness of fit of the model was examined using the Hosmer-Lemeshow (non-significant p-value indicating good fit) [23]. All statistical analyses were done using R version 3.4.0 [24].

## Results

### Study population and response

**Fig 1:**
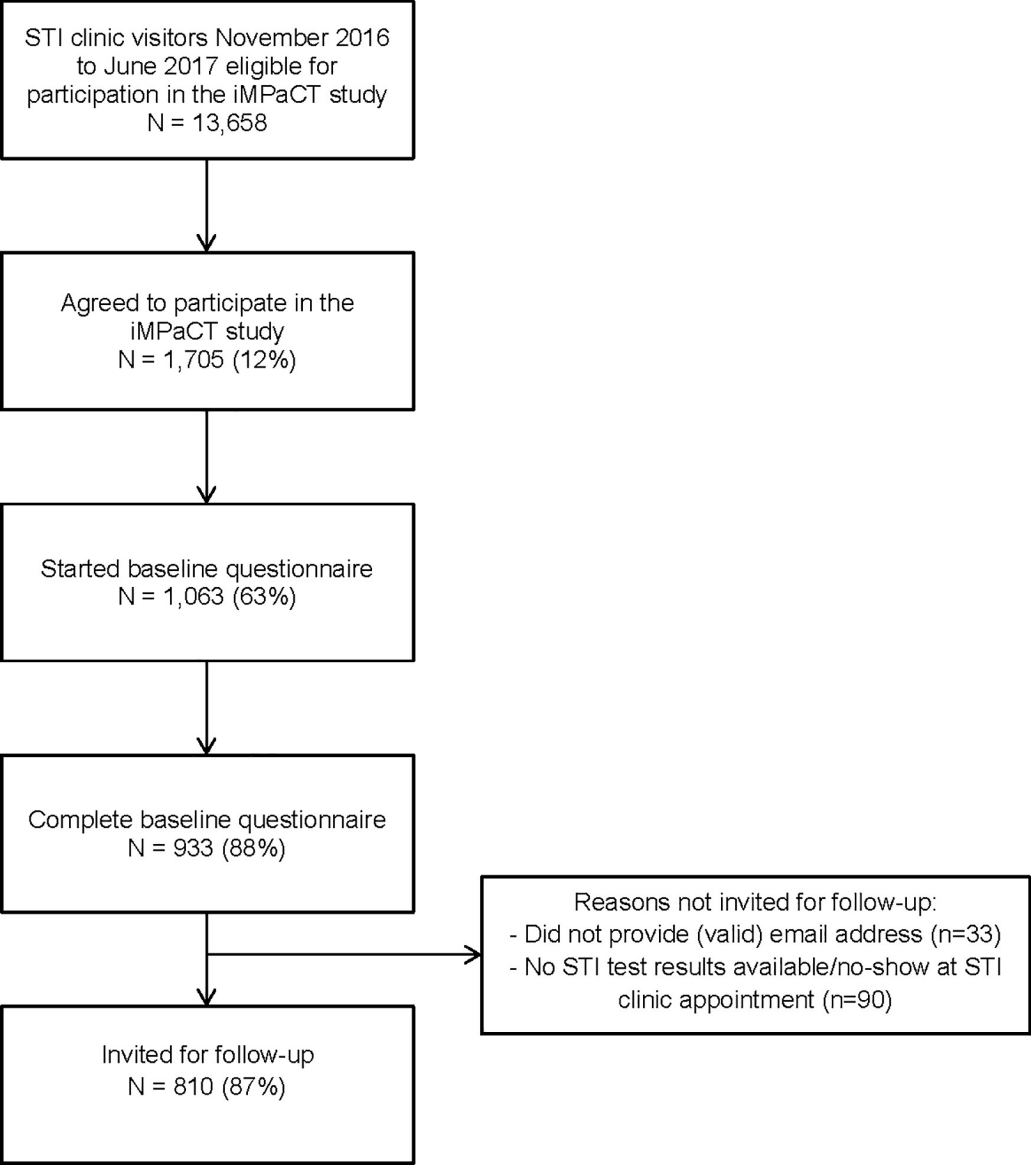
Baseline response rates in the iMPaCT study. Abbreviations: STI = Sexually Transmitted Infection.

In total, 13,658 STI clinic visitors were eligible to participate in the iMPaCT study. The majority of those STI clinic visitors was ≥ 21 years (76%), female (72%), highly educated (71%), and ethnic Dutch (72%). Of those STI clinic visitors, 2,253 (16%) actively declined the online invitation, 1,705 (12%) agreed to participate, and 1,063 (8%) started the online questionnaire, and 933 (7%) completed the baseline questionnaire (Fig 1). The majority of the participants was ≥23 years old (52%), female (81%), highly educated (89%), and ethnic Dutch (81%). Furthermore, 79% of the participants were recruited at the STI clinic in Amsterdam (n = 838), 7% in Kennemerland (n = 81), 10% in Hollands Noorden (n = 105), and 4% in Twente (n = 39), resulting in response rates of 9%, 6%, 7%, and 3% respectively.

**Fig 2:**
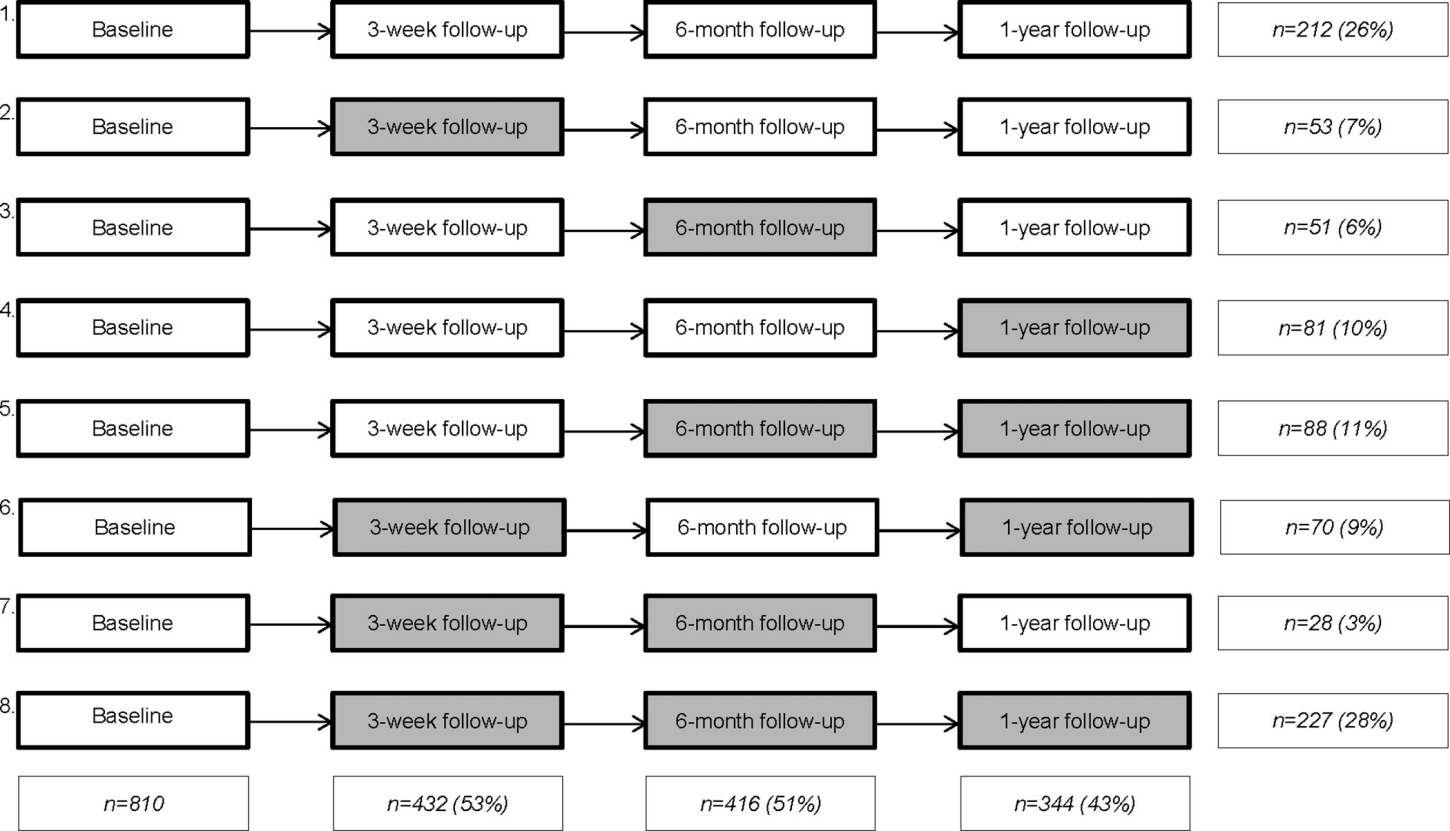
Response participants invited for follow-up, at three-week, six-month, and one-year follow-up. Grey indicates (partial) lost to follow-up.

Of all participants who completed the baseline questionnaire), 810 participants could be invited to participate in the follow-up data collection moments. Of these 810 participants, 432 (53%) filled out the online questionnaire at three-week follow-up, 416 (51%) filled out the online questionnaire at six-month follow-up, and 344 (43%) filled out the last questionnaire at one-year follow-up (Fig 2). Furthermore, 26% of the participants completed all three follow-up questionnaires, 23% completed two follow-up questionnaires, 23% completed one follow-up questionnaire, and 28% did not respond to any of the follow-up questionnaires. All 416 participants who filled out the questionnaire at six-month follow-up received a home-based test kit, and 315 (76%) send the test kit to the laboratory for chlamydia and gonorrhea testing.

### Predictors for non-response at baseline

All demographic, behavioral and sexual health-related variables were significant predictors of non-response in the univariable analysis (Table 1). In the multivariable analysis, type of STI test was highly correlated to other explanatory variables as type of STI test is dependent on a number of predictors in the model (i.e., triage criteria, such as age, and migration background), and was therefore excluded from the model. In the final multivariable model, there was no multicollinearity (VIF values around 1), and the Hosmer-Lemeshow test indicated good fit (p-value = 0.3). Male gender, low/medium education level, young age (≤ 20 years), and being non-Dutch, were significant predictors of non-response at baseline. Furthermore, non-respondents were more likely to report STI-related symptoms, being notified by a partner, ≤ 2 partners in the past six months, and having had condomless sex at the last sex act compared to the participants. The chlamydia positivity rate at baseline was significantly higher (p = 0.003) in the non-respondents (n = 2,153, 17%) compared to the participants (n = 143, 14%).

**Table 1:** Univariable and multivariable logistic regression analysis of predictors for non-response in the iMPaCT study by comparing participants (n = 1,063) and non-respondents (n = 12,595) aged 18–24 years visiting an STI clinic in November 2016 to June 2017.

	*Participants*		*Non-respondents*			
	N	%	N	%	*OR (95% CI)*	*aOR (95%CI)*
Age						
18–20 years	165	16	3057	24	1	1
21–22 years	342	32	4567	36	**0.72 (0.59–0.87)**	**0.79 (0.65–0.96)**
23–24 years	556	52	4971	40	**0.48 (0.40–0.58)**	**0.54 (0.45–0.65)**
Gender						
Female	860	81	9018	72	1	1
Male	203	19	3577	28	**1.68 (1.44–1.97)**	**1.73 (1.47–2.05)**
Education level						
Low/medium	120	11	3563	28	1	1
High	941	89	8692	69	**0.31 (0.26–0.38)**	**0.40 (0.32–0.48)**
Migration background						
Ethnic Dutch	857	81	8955	71	1	1
Non-Dutch	206	19	3638	29	**1.69 (1.45–1.98)**	**1.27 (1.08–1.49)**
Symptoms						
No	897	84	9649	77	1	1
Yes	166	16	2946	23	**1.65 (1.39–1.96)**	**1.35 (1.14–1. 62)**
GO/CT/SYPH past year						
No	320	30	3092	24	1	-
Yes	129	12	1603	13	**1.29 (1.04–1.60)**	-
Not tested	614	58	7900	63	**1.33 (1.15–1.53)**	-
Partner notification						
No	942	89	10684	85	1	1
Yes	121	11	1910	15	**1.39 (1.15–1.70)**	**1.23 (1.01–1.51)**
Number of partners in past six months						
0–2 partners	374	35	6005	48	1	1
3–4 partners	396	37	3956	31	**0.62 (0.54–0.72)**	**0.66 (0.57–0.77)**
≥ 5 partners	293	28	2634	21	**0.56 (0.48–0.66)**	**0.55 (0.47–0.65)**
Condom use at last sexual contact						
No	828	78	10072	80	1	1
Yes	228	22	2386	19	*0*.*86 (0*.*74–1*.*00)*	**0.83 (0.71–0.98)**

Footnote: Categories do not all add up to 100%, as missing values are not shown. Statistical associations are shown in in italic when the p-value is equal to or smaller than 0.1, and in bold when the p-value is equal to or smaller than 0.05.

Abbreviations: CT = Chlamydia; OR = crude odds ratio, aOR = adjusted odds ratio; CI = Confidence Interval; IC = Informed Consent; STI = Sexually Transmitted Infection; SYPH = Syphilis.

### Predictors for loss to follow-up

Interactions were found between STI clinic region (Amsterdam/non-Amsterdam) and the behavioral and psychological predictors. For these predictors, the analyses were stratified by STI region. Low/medium health goals at baseline was a significant predictor of non-response at six-month, and/or one-year follow-up in both Amsterdam (Table 2) and non-Amsterdam region (Table 3). Predictors of loss to follow-up in Amsterdam were low/medium social norms and support (at three-week follow-up), and having had ≥ 5 partners in the past six months (at six-month follow-up). For non-Amsterdam, high impulsiveness (at six-month follow-up), and high intentions (at one-year follow-up) were predictors of loss to follow-up. High risk perception at baseline in Amsterdam, and low/medium risk perception at baseline in non-Amsterdam was a significant predictor of loss to follow-up at six-month and one-year.

**Table 2:** Multivariable logistic regression analyses of predictors of non-response at the three follow-up data collection moments for participants who visited the STI clinic in Amsterdam.

	*Baseline*		*3-week follow-up non-response*			*6-month follow-up non-response*			*1-year follow-up non-response*		
	N	%	N	%	*aOR*	N	%	*aOR*	N	%	*aOR*
					*(95%CI)*			*(95%CI)*			*(95%CI)*
Total	647		292	45		303	47		365	56	
Number of partners in past six months											
0–2 partners	222	34	94	32	-	93	31	1	117	32	-
3–4 partners	261	40	123	42	-	114	38	0.99 (0.68–1.43)	143	39	-
≥ 5 partners	164	25	75	26	-	96	32	**1.73 (1.14–2.64)**	105	29	-
Condom use at last sex act											
No	504	78	235	81	-	239	79	-	300	82	1
Yes	143	22	57	20	-	64	21	-	65	18	*0*.*70 (0*.*47–1*.*03)*
Age at sexual debut											
< 16 years	205	32	102	35	-	100	33	-	123	34	-
≥ 16 years	442	68	190	65	-	203	67	-	242	66	-
Health goals											
Low/med (score < 4.00)	314	49	159	55	1	168	55	1	200	55	1
High (score ≥ 4.00)	333	52	133	46	0.77 (0.55–1.07)	135	45	**0.65 (0.47–0.90)**	165	45	**0.65 (0.47–0.90)**
Attitudes*											
Low/med (score < 4.25)	265	41	136	47	1	140	46	-	167	46	-
High (score ≥ 4.25)	382	59	156	53	0.76 (0.54–1.06)	163	54	-	198	54	-
Social norms and support											
Low/med (score < 3.20)	229	35	122	42	1	119	39	-	140	38	-
High (score ≥ 3.20)	418	65	170	58	**0.65 (0.47–0.91)**	184	61	-	225	62	-
Risk perception for CT (own risk)											
Low/med (score < 27.50)	311	48	134	46	-	126	42	1	151	41	1
High (score ≥ 27.50)	336	52	158	54	-	177	58	**1.51 (1.09–2.08)**	214	59	**1.70 (1.23–2.33)**

* Attitudes regarding prevention of chlamydia

Footnote: Categories do not all add up to 100%, as missing values are not shown. Statistical associations are shown in in italic when the p-value is equal to or smaller than 0.1, and in bold when the p-value is equal to or smaller than 0.05. Only variables that were pre-selected in the univariable analyses are shown here.

Abbreviations: aOR = adjusted odds ratio, CI = Confidence Interval; Low/med = Low/medium, CT = Chlamydia; STI = Sexually Transmitted Infection.

**Table 3:** Multivariable logistic regression analyses of predictors of non-response at the three follow-up data collection moments for participants who visited the STI clinics in Kennemerland, Hollands Noorden, and Twente (non-Amsterdam).

	*Baseline*		*3-week follow-up non-response*			*6-month follow-up non-response*			*1-year follow-up non-response*		
	N	%	N	%	*aOR*	N	%	*aOR*	N	%	*aOR*
					*(95%CI)*			*(95%CI)*			*(95%CI)*
Total	163		86	53		91	56		101	62	
Health goals											
Low/med (score < 4.00)	83	51	49	57	1	49	54	-	57	56	1
High (score ≥ 4.00)	80	49	37	43	*0*.*58 (0*.*31–1*.*08)*	42	46	-	44	44	**0.40 (0.18–0.75)**
Intentions											
Low/med (score < 2.67)	84	52	44	51	-	45	50	-	46	46	1
High (score ≥ 2.67)	79	48	42	49	-	46	51	-	55	55	**2.16 (1.08–4.44)**
Impulsiveness											
Low/med (score < 2.63)	78	48	39	45	-	35	39	1	45	45	-
High (score ≥ 2.63)	85	52	47	55	-	56	62	**2.97 (1.52–5.99)**	56	55	-
Knowledge*											
Low/med (score < 6.00)	91	56	48	56	**-**	56	62	1	60	59	-
High (score ≥ 6.00)	72	44	38	44	**-**	35	39	0.58 (0.30–1.12)	41	41	-
Risk perception for CT (own risk)											
Low/med (score < 27.50)	84	52	44	51	-	52	57	1	58	57	1
High (score ≥ 27.50)	79	48	42	49	-	39	43	**0.45 (0.23–0.88)**	43	43	**0.47 (0.23–0.94)**

* Knowledge regarding sexual health, prevention of chlamydia and consequences of chlamydia diagnosis

Footnote: Categories do not all add up to 100%, as missing values are not shown. Statistical associations are shown in in italic when the p-value is equal to or smaller than 0.1, and in bold when the p-value is equal to or smaller than 0.05. Only variables that were pre-selected in the univariable analyses are shown here.

Abbreviations: aOR = adjusted odds ratio, CI = Confidence Interval; Low/med = Low/medium, CT = Chlamydia; STI = Sexually Transmitted Infection.

Stratified multivariable analyses by STI clinic region using demographic and sexual health-related predictors was not possible due to the small number of observations in each cell (S1 and S2 Tables), and these predictors were analyzed separately without stratification. Male gender, low/medium education level, and younger age (≤ 20 years) were associated with non-response at either three-week, six-month and/or one-year follow-up (S3 Table). Chlamydia infection at baseline was not a predictor of loss to follow-up.

## Discussion

Our results showed that male, younger age (≤ 20 years), and low/medium educated individuals were more likely to be non-respondents at baseline and more likely to be lost to follow-up after baseline participation. Furthermore, behavioral and psychological variables appeared to play a role in non-response at long-term follow-up. Behavioral and psychological predictors of loss to follow-up were different between STI clinic regions, except for low perceived importance of health at baseline, which was predictive of loss to follow-up at six-month and one-year follow-up in all STI clinic regions. The chlamydia positivity rate was significantly higher among non-respondents than among the participants, but chlamydia infection itself at baseline was not a predictor of loss to follow-up.

The main strength of this study is the comprehensive data consisting of behavioral characteristics, sexual health outcomes and demographic characteristics on both participants and non-respondents. Moreover, to our knowledge, this is the first study identifying psychological predictors for loss to follow-up among both chlamydia diagnosed and undiagnosed heterosexual STI clinic visitors. Furthermore, the extensive non-response analysis provided insights into potential bias and generalizability of the study population. This study was, however, not without some limitations. First, reasons for non-response at baseline or loss to follow-up were not recorded. Nevertheless, reasons for non-response in health research, such as lack of time, being forgetful, or privacy concerns [10, 25–27], are well documented, and were not the purpose of this study. Second, the actual number of eligible STI clinic visitors who were invited for participation during the recruitment period was not known. As recruitment was only done through the online registration form at the STI clinics in Amsterdam, Kennemerland and Hollands Noorden, individuals who booked an appointment via telephone were not invited for participation (4%-17% of all eligible STI clinic visitors (M.S. van Rooijen, personal communication, October 17, 2018). At the STI clinic in Twente, no online registration exists, and recruitment was only done when people booked an appointment via telephone. However, not all eligible participants were invited due to lack of time or forgetting or omitting to inform eligible STI clinic visitors, but we were not able to distinguish invitees from non-invitees, meaning that actual response rates are higher.

We found that male gender, lower education, and a lower number of sexual partners were predictors of non-response, which was consistent with the literature [3, 5, 11, 28–30]. Male gender, and lower education level were, as well as being predictors of non-response at baseline, also predictors of loss to follow-up, which is also in line with findings from previous studies [17, 31]. In contrast to other STI studies, we found that individuals who reported STI-related symptoms were less likely to participate at baseline. This might be explained by the differences in the study design. Participants in two other Dutch STI studies [5, 30], received a free chlamydia test kit if they agreed to participate, which might have provided extra motivation for individuals with STI-related symptoms to participate. In our study, participation was not required to receive the first STI test, as individuals who were invited to participate had already made an appointment for an STI test at the clinic.

We found that chlamydia positivity rates were significantly higher in non-respondents compared to participants at baseline, while individuals reporting a higher number of partners in the past six months, (classically categorized as high-risk [32, 33]), participated more than individuals reporting lower number of partners. A possible explanation for this contradiction might be that non-respondents, although having fewer partners, more often reported condomless sex at last sexual contact, STI-related symptoms, and being notified by their partner, which are also known risk factors for chlamydia infection [34]. Condomless sex at last sexual contact should, however, be interpreted with caution, as reporting condomless sex in a monogamous relationship does not necessarily reflect higher chlamydia risk [35].

Low perceived importance of health at baseline was a predictor of long-term loss to follow-up (at six-month and one-year follow-up) in all STI clinic regions. This finding might be related to earlier findings that showed that long-term health goals might influence certain behaviors, such as participation behavior (i.e., motivation to participate in health research) [36, 37]. In non-Amsterdam, high intentions towards condom use and STI testing at baseline and high impulsiveness were predictors of loss to follow-up, and in Amsterdam, less positive attitudes regarding prevention of chlamydia and lower social norms and support were associated with loss to follow-up. These results partly reflect the theory of planned behavior, that links attitudes and social norms to intended behavior [38], and with previous studies that found that individuals are not always able to carry out intended behavior [39–41]. Low perceived risk of chlamydia at baseline was associated with loss to follow-up at six-month and one-year follow-up in non-Amsterdam, which is in line with the health belief model (i.e., perceived seriousness/susceptibility associated with likelihood of engaging in behavior [42]) and has previously been described as the main reason for non-response [10]. However, in Amsterdam, high risk perception was a predictor of loss to follow-up. A possible explanation for this contradictory finding is that we examined the association between risk perception at baseline and loss to follow-up one year later, and not risk perception after one year. It might be that risk perception decreased after baseline in Amsterdam, because individuals believed they were engaging in less risky sexual behavior than before or they believed they overestimated their risk at baseline [43]. Lower risk perception at the follow-up moments might have negatively influenced the motivation to participate in the follow-up moments.

The findings of this study indicate that to prevent non-response bias, recruitment strategies should put more effort into recruiting underrepresented behavioral and demographic groups in sexual health-related research. For example, targeted recruitment or cultural adaptations (e.g., flyers and/or personalized invitations adapted to migrants' culture and language) [44], or adapting the recruitment method to increase interest and motivation among males (e.g., raising awareness or a greater sense of responsibility in terms of health in males) [29], might be effective in improving response rates in these underrepresented groups. Furthermore, the psychological predictors of loss to follow-up identified in this study could also be used as potential targets for recruitment strategies to increase retention. Recruitment strategies focusing on increasing perceived importance of health (i.e., health goals) by stimulating (health) goal pursuit [36, 37], or improving perceived risk [45] using risk communication targeting different elements of risk perception simultaneously (e.g., perceived severity and self-efficacy) [46], might increase response rates and retention in sexual health-related research. Moreover, implementation intentions (i.e., formulating a specific plan or *implementation intention*) might be an effective strategy to improve participation behavior, as it targets a variety of factors related to (intended) behavior [39], including the different psychological predictors of loss to follow-up identified in this study. Future research should be undertaken to investigate the impact of targeted recruitment strategies on response and retention in underrepresented demographic, behavioral, and psychological groups.

In conclusion, differences in demographic, behavioral, and psychological characteristics need to be taken into consideration in recruitment strategies. Tailoring recruitment strategies to demographic characteristics, behavioral, and psychological characteristics, might be needed to increase response rates and retention, and to prevent non-response bias in health research.

## Supporting information

S1 Table Univariable logistic regression analyses of predictors of non-response at the three follow-up data collection moments for participants who visited the STI clinic in Amsterdam. (DOCX)

S2 Table Univariable logistic regression analyses of predictors of non-response at the three follow-up data collection moments for participants who visited the STI clinics in Kennemerland, Hollands Noorden, and Twente (non-Amsterdam). (DOCX)

S3 Table Multivariable logistic regression analyses of demographic and sexual health-related predictors of non-response at the three follow-up data collection moments. (DOCX)

S1 File iMPaCT Questionnaire (Dutch). (PDF)

S2 File iMPaCT Questionnaire (English). (PDF)
