# Supplementary material for: Who drops out and when? Predictors of non-response and loss to follow-up in a longitudinal cohort study among STI clinic visitors
Source: PLoS One. 2019 Jun 19;14(6):e0218658. doi: 10.1371/journal.pone.0218658 (PMC6583983; doi:10.1371/journal.pone.0218658)
Supplement: S1 Table — (DOCX) [file pone.0218658.s001.docx]

**S1 Table. Univariable logistic regression analyses of predictors of non-response at the three follow-up data collection moments for participants who visited the STI clinic in Amsterdam**

|  | *Baseline* | | *3-week follow-up non-response* | | | *6-month follow-up non-response* | | | *1-year follow-up non-response* | | |
| --- | --- | --- | --- | --- | --- | --- | --- | --- | --- | --- | --- |
|  | N | % | N | % | *OR* | N | % | *OR* | N | % | *OR* |
|  |  |  |  |  | *(95%CI)* |  |  | *(95%CI)* |  |  | *(95%CI)* |
| Total | 647 |  | 292 | 45 |  | 303 | 47 |  | 365 | 56 |  |
| Age |  |  |  |  |  |  |  |  |  |  |  |
| 18-20 years | 135 | 21 | 65 | 22 | 1 | 77 | 25 | 1 | 83 | 23 | 1 |
| 21-22 years | 233 | 36 | 108 | 37 | 0.94 (0.61-1.44) | 114 | 38 | 0.72 (0.47-1.09) | 132 | 36 | 0.83 (0.53-1.27) |
| 23-24 years | 279 | 43 | 119 | 41 | 0.80 (0.53-1.21) | 112 | 37 | **0.51 (0.33-0.76)** | 150 | 41 | 0.73 (0.48-1.10) |
| Gender |  |  |  |  |  |  |  |  |  |  |  |
| Female | 525 | 81 | 222 | 76 | 1 | 228 | 75 | 1 | 277 | 76 | 1 |
| Male | 122 | 19 | 70 | 24 | **1.83 (1.23-2.74)** | 75 | 25 | **2.09 (1.40-3.14)** | 88 | 24 | **2.31 (1.52-3.59)** |
| Education level |  |  |  |  |  |  |  |  |  |  |  |
| Low/med | 47 | 7 | 23 | 8 | 1 | 26 | 9 | 1 | 34 | 9 | 1 |
| High | 599 | 93 | 269 | 92 | 0.85 (0.47-1.55) | 276 | 91 | 0.69 (0.38-1.25) | 331 | 91 | **0.47 (0.24-0.89)** |
| Migration background |  |  |  |  |  |  |  |  |  |  |  |
| Dutch | 508 | 79 | 225 | 77 | 1 | 228 | 75 | 1 | 276 | 76 | 1 |
| Non-Dutch | 139 | 22 | 67 | 23 | 1.17 (0.90-1.70) | 75 | 25 | *1.45 (0.99-2.11)* | 89 | 24 | **1.49 (1.01-2.21)** |
| Symptoms |  |  |  |  |  |  |  |  |  |  |  |
| No | 561 | 87 | 255 | 87 | 1 | 255 | 84 | 1 | 320 | 88 | 1 |
| Yes | 86 | 13 | 37 | 13 | 0.90 (0.57-1.42) | 48 | 16 | *1.52 (0.97-2.41)* | 45 | 12 | 0.82 (0.52-1.30) |
| GO/CT/SYPH past year |  |  |  |  |  |  |  |  |  |  |  |
| No | 205 | 32 | 98 | 34 | 1 | 90 | 30 | 1 | 118 | 32 | 1 |
| Yes | 78 | 12 | 36 | 12 | 0.94 (0.55-1.58) | 40 | 13 | 1.35 (0.80-2.27) | 45 | 12 | 1.01 (0.60-1.71) |
| Not tested | 364 | 56 | 158 | 54 | 0.84 (0.60-1.19) | 173 | 57 | 1.15 (0.82-1.63) | 202 | 55 | 0.93 (0.65-1.31) |
| Partner notification |  |  |  |  |  |  |  |  |  |  |  |
| No | 569 | 88 | 251 | 86 | 1 | 267 | 88 | 1 | 319 | 87 | 1 |
| Yes | 78 | 12 | 41 | 14 | 1.40 (0.87-2.26) | 36 | 12 | 0.97 (0.60-1.56) | 46 | 13 | 1.12 (0.70-1.83) |
| Number of partners in past six months |  |  |  |  |  |  |  |  |  |  |  |
| 0-2 partners | 222 | 34 | 94 | 32 | 1 | 93 | 31 | 1 | 117 | 32 | 1 |
| 3-4 partners | 261 | 40 | 123 | 42 | 1.20 (0.84-1.73) | 114 | 38 | 1.09 (0.76-1.56) | 143 | 39 | 1.08 (0.75-1.54) |
| ≥ 5 partners | 164 | 25 | 75 | 26 | 1.14 (0.76-1.71) | 96 | 32 | **1.98 (1.32-2.99)** | 105 | 29 | **1.58 (1.05-2.40)** |
| Condom use at last sexual contact |  |  |  |  |  |  |  |  |  |  |  |
| No | 504 | 78 | 235 | 81 | 1 | 239 | 79 | 1 | 300 | 82 | 1 |
| Yes | 143 | 22 | 57 | 20 | 0.77 (0.52-1.12) | 64 | 21 | 0.88 (0.61-1.28) | 65 | 18 | **0.57 (0.39-0.83)** |
| Age at sexual debut |  |  |  |  |  |  |  |  |  |  |  |
| < 16 years | 205 | 32 | 102 | 35 | 1 | 100 | 33 | 1 | 123 | 34 | 1 |
| ≥ 16 years | 442 | 68 | 190 | 65 | *0.75 (0.54-1.05)* | 203 | 67 | 0.90 (0.65-1.26) | 242 | 66 | 0.80 (0.57-1.11) |
| Health goals |  |  |  |  |  |  |  |  |  |  |  |
| Low/med (score < 4.00) | 314 | 49 | 159 | 55 | 1 | 168 | 55 | 1 | 200 | 55 | 1 |
| High (score ≥ 4.00) | 333 | 52 | 133 | 46 | **0.65 (0.48-0.89)** | 135 | 45 | **0.59 (0.43-0.80)** | 165 | 45 | **0.56 (0.41-0.77)** |
| Attitudes^a^ |  |  |  |  |  |  |  |  |  |  |  |
| Low/med (score < 4.25) | 265 | 41 | 136 | 47 | 1 | 140 | 46 | 1 | 167 | 46 | 1 |
| High (score ≥ 4.25) | 382 | 59 | 156 | 53 | **0.66 (0.48-0.90)** | 163 | 54 | **0.66 (0.48-0.90)** | 198 | 54 | **0.63 (0.46-0.87)** |
| Intentions |  |  |  |  |  |  |  |  |  |  |  |
| Low/med (score < 2.67) | 391 | 60 | 171 | 59 | 1 | 181 | 60 | 1 | 228 | 63 | 1 |
| High (score ≥ 2.67) | 256 | 40 | 121 | 41 | 1.16 (0.85-1.60) | 122 | 40 | 1.05 (0.76-1.44) | 137 | 38 | 0.83 (0.60-1.14) |
| Anticipated stigma |  |  |  |  |  |  |  |  |  |  |  |
| Low/med (score < 2.17) | 297 | 46 | 138 | 47 | 1 | 133 | 44 | 1 | 170 | 47 | 1 |
| High (score ≥ 2.17) | 350 | 54 | 154 | 53 | 0.91 (0.67-1.24) | 170 | 56 | 1.16 (0.85-1.58) | 195 | 53 | 0.95 (0.69-1.29) |
| Anticipated shame |  |  |  |  |  |  |  |  |  |  |  |
| Low/med (score < 3.75) | 257 | 40 | 120 | 41 | 1 | 127 | 42 | 1 | 147 | 40 | 1 |
| High (score ≥ 3.75) | 390 | 60 | 172 | 59 | 0.90 (0.66-1.24) | 176 | 58 | 0.84 (0.61-1.15) | 218 | 60 | 0.95 (0.69-1.31) |
| Impulsiveness |  |  |  |  |  |  |  |  |  |  |  |
| Low/med (score < 2.63) | 307 | 47 | 136 | 47 | 1 | 143 | 47 | 1 | 163 | 45 | 1 |
| High (score ≥ 2.63) | 340 | 53 | 156 | 53 | 1.06 (0.78-1.45) | 160 | 53 | 1.03 (0.75-1.40) | 202 | 55 | 1.38 (0.94-1.76) |
| Social norms and support |  |  |  |  |  |  |  |  |  |  |  |
| Low/med (score < 3.20) | 229 | 35 | 122 | 42 | 1 | 119 | 39 | 1 | 140 | 38 | 1 |
| High (score ≥ 3.20) | 418 | 65 | 170 | 58 | 0.60 (0.44-0.83) | 184 | 61 | **0.72 (0.52-1.00)** | 225 | 62 | *0.75 (0.54-1.03)* |
| Knowledge^b^ |  |  |  |  |  |  |  |  |  |  |  |
| Low/med (score < 6.00) | 285 | 44 | 136 | 47 | 1 | 143 | 47 | 1 | 156 | 43 | 1 |
| High (score ≥ 6.00) | 362 | 56 | 156 | 53 | 0.83 (0.61-1.14) | 160 | 53 | 0.78 (0.57-1.07) | 209 | 57 | 1.14 (0.83-1.56) |
| Self-efficacy |  |  |  |  |  |  |  |  |  |  |  |
| Low/med (score < 3.00) | 330 | 51 | 159 | 55 | 1 | 154 | 51 | 1 | 190 | 52 | 1 |
| High (score ≥ 3.00) | 317 | 49 | 133 | 46 | 0.78 (0.57-1.07) | 149 | 49 | 1.01 (0.74-1.37) | 175 | 48 | 0.91 (0.67-1.25) |
| Self-esteem |  |  |  |  |  |  |  |  |  |  |  |
| Low/med (score < 3.74) | 200 | 31 | 90 | 31 | 1 | 93 | 31 | 1 | 111 | 30 | 1 |
| High (score ≥ 3.74) | 447 | 69 | 202 | 69 | 1.01 (0.72-1.42) | 210 | 69 | 1.01 (0.73-1.42) | 254 | 70 | 1.06 (0.76-1.48) |
| Risk perception for CT (own risk) |  |  |  |  |  |  |  |  |  |  |  |
| Low/med (score < 27.50) | 311 | 48 | 134 | 46 | 1 | 126 | 42 | 1 | 151 | 41 | 1 |
| High (score ≥ 27.50) | 336 | 52 | 158 | 54 | 1.17 (0.85-1.59) | 177 | 58 | **1.65 (1.21-2.25)** | 214 | 59 | **1.85 (1.35-2.53)** |
| CT infection |  |  |  |  |  |  |  |  |  |  |  |
| No | 570 | 88 | 257 | 88 | 1 | 269 | 89 | 1 | 322 | 88 | 1 |
| Yes | 77 | 12 | 35 | 12 | 1.04 (0.64-1.68) | 34 | 11 | 0.86 (0.53-1.39) | 43 | 12 | 1.00 (0.62-1.64) |

^a^ Attitudes regarding prevention of chlamydia

^b^ Knowledge regarding sexual health, prevention of chlamydia and consequences of chlamydia diagnosis

Footnote: Categories do not all add up to 100%, as missing values are not shown. Statistical associations are shown in in italic when the p-value is equal to or smaller than 0.1, and in bold when the p-value is equal to or smaller than 0.05.

Abbreviations: OR = crude odds ratio, CI = Confidence Interval; Low/med = Low/medium, CT = Chlamydia; STI = Sexually Transmitted Infection.
