# Supplementary material for: Who drops out and when? Predictors of non-response and loss to follow-up in a longitudinal cohort study among STI clinic visitors
Source: PLoS One. 2019 Jun 19;14(6):e0218658. doi: 10.1371/journal.pone.0218658 (PMC6583983; doi:10.1371/journal.pone.0218658)
Supplement: S2 Table — (DOCX) [file pone.0218658.s002.docx]

**S2 Table. Univariable logistic regression analyses of predictors of non-response at the three follow-up data collection moments for participants who visited the STI clinics in Kennemerland, Hollands Noorden, and Twente (non-Amsterdam)**

|  | *Baseline* | | *3-week follow-up non-response* | | | *6-month follow-up non-response* | | | *1-year follow-up non-response* | | |
| --- | --- | --- | --- | --- | --- | --- | --- | --- | --- | --- | --- |
|  | N | % | N | % | *OR* | N | % | *OR* | N | % | *OR* |
|  |  |  |  |  | *(95%CI)* |  |  | *(95%CI)* |  |  | *(95%CI)* |
| Total | 163 |  | 86 | 53 |  | 91 | 56 |  | 101 | 62 |  |
| Age |  |  |  |  |  |  |  |  |  |  |  |
| 18-20 years | 44 | 27 | 21 | 24 | 1 | 22 | 24 | 1 | 25 | 25 | 1 |
| 21-22 years | 64 | 39 | 35 | 41 | 1.32 (0.61-2.87) | 39 | 43 | 1.56 (0.72-3.41) | 43 | 43 | 1.56 (0.70-3.46) |
| 23-24 years | 55 | 34 | 30 | 35 | 1.27 (0.57-2.84) | 30 | 33 | 1.16 (0.52-2.59) | 33 | 33 | 1.11 (0.49-2.48) |
| Gender* |  |  |  |  |  |  |  |  |  |  |  |
| Female | 126 | 77 | 62 | 72 | 1 | 65 | 71 | 1 | 71 | 70 | 1 |
| Male | 37 | 23 | 24 | 28 | *1.94 (0.92-4.24)* | 26 | 29 | **1.94 (0.92-4.24)** | 30 | 30 | **3.37 (1.44-8.86)** |
| Education level* |  |  |  |  |  |  |  |  |  |  |  |
| Low/med | 37 | 23 | 25 | 29 | 1 | 27 | 30 | 1 | 27 | 27 | 1 |
| High | 126 | 77 | 61 | 71 | *0.47 (0.21-1.00)* | 64 | 70 | **0.40 (0.17-0.87)** | 74 | 73 | 0.55 (0.23-1.20) |
| Migration background |  |  |  |  |  |  |  |  |  |  |  |
| Dutch | 148 | 91 | 78 | 91 | 1 | 81 | 89 | 1 | 91 | 90 | 1 |
| Non-Dutch | 15 | 9 | 8 | 9 | 1.04 (0.36-3.10) | 10 | 11 | 1.68 (0.57-5.60) | 10 | 10 | 1.27 (0.43-4.24) |
| Symptoms |  |  |  |  |  |  |  |  |  |  |  |
| No | 123 | 76 | 64 | 74 | 1 | 66 | 73 | 1 | 77 | 76 | 1 |
| Yes | 40 | 25 | 22 | 26 | 1.14 (0.60-2.36) | 25 | 28 | 1.46 (0.71-3.09) | 24 | 24 | 0.91 (0.44-1.91) |
| GO/CT/SYPH past year |  |  |  |  |  |  |  |  |  |  |  |
| No | 40 | 25 | 20 | 23 | 1 | 24 | 26 | 1 | 22 | 22 | 1 |
| Yes | 19 | 12 | 13 | 15 | 2.28 (0.74-7.64) | 9 | 10 | 0.63 (0.20-1.89) | 11 | 11 | 1.18 (0.39-3.65) |
| Not tested | 104 | 64 | 53 | 62 | 1.09 (0.52-2.30) | 58 | 64 | 0.88 (0.41-1.84) | 68 | 67 | 1.62 (0.76-3.43) |
| Partner notification* |  |  |  |  |  |  |  |  |  |  |  |
| No | 147 | 90 | 81 | 94 | 1 | 85 | 93 | 1 | 93 | 92 | 1 |
| Yes | 16 | 10 | 5 | 6 | *0.38 (0.11-1.07)* | 6 | 7 | 0.44 (0.14-1.26) | 8 | 8 | 0.59 (0.20-1.68) |
| Number of partners in past six months |  |  |  |  |  |  |  |  |  |  |  |
| 0-2 partners | 75 | 46 | 40 | 47 | 1 | 43 | 47 | 1 | 52 | 52 | 1 |
| 3-4 partners | 46 | 28 | 26 | 30 | 1.09 (0.52-2.31) | 24 | 26 | 0.78 (0.37-1.64) | 26 | 26 | 0.55 (0.26-1.19) |
| ≥ 5 partners | 42 | 26 | 20 | 23 | 0.80 (0.37-1.70) | 24 | 26 | 0.99 (0.46-2.14) | 23 | 23 | 0.54 (0.24-1.17) |
| Condom use at last sexual contact* |  |  |  |  |  |  |  |  |  |  |  |
| No | 120 | 74 | 66 | 77 | 1 | 70 | 77 | 1 | 79 | 78 | 1 |
| Yes | 42 | 26 | 19 | 22 | 0.68 (0.33-1.37) | 20 | 22 | 0.65 (0.32-1.32) | 21 | 21 | *0.52 (0.25-1.06)* |
| Age at sexual debut |  |  |  |  |  |  |  |  |  |  |  |
| < 16 years | 67 | 41 | 37 | 43 | 1 | 39 | 43 | 1 | 42 | 42 | 1 |
| ≥ 16 years | 96 | 59 | 49 | 57 | 0.87 (0.46-1.63) | 52 | 57 | 0.87 (0.46-1.64) | 59 | 58 | 0.97 (0.51-1.85) |
| Health goals |  |  |  |  |  |  |  |  |  |  |  |
| Low/med (score < 4.00) | 83 | 51 | 49 | 57 | 1 | 49 | 54 | 1 | 57 | 56 | 1 |
| High (score ≥ 4.00) | 80 | 49 | 37 | 43 | *0.58 (0.31-1.08)* | 42 | 46 | 0.75 (0.40-1.39) | 44 | 44 | *0.54 (0.28-1.03)* |
| Attitudes^a^ |  |  |  |  |  |  |  |  |  |  |  |
| Low/med (score < 4.25) | 73 | 45 | 42 | 49 | 1 | 45 | 50 | 1 | 50 | 50 | 1 |
| High (score ≥ 4.25) | 90 | 55 | 44 | 51 | 0.69 (0.37-1.28) | 46 | 51 | 0.64 (0.34-1.19) | 51 | 51 | 0.59 (0.31-1.12) |
| Intentions |  |  |  |  |  |  |  |  |  |  |  |
| Low/med (score < 2.67) | 84 | 52 | 44 | 51 | 1 | 45 | 50 | 1 | 46 | 46 | 1 |
| High (score ≥ 2.67) | 79 | 48 | 42 | 49 | 1.01 (0.54-1.87) | 46 | 51 | 1.18 (0.64-2.21) | 55 | 55 | *1.86 (0.98-3.57)* |
| Anticipated stigma |  |  |  |  |  |  |  |  |  |  |  |
| Low/med (score < 2.17) | 67 | 41 | 36 | 42 | 1 | 39 | 43 | 1 | 45 | 45 | 1 |
| High (score ≥ 2.17) | 96 | 59 | 50 | 58 | 0.96 (0.51 -1.80) | 52 | 57 | 0.87 (0.46-1.64) | 56 | 55 | 0.70 (0.36-1.34) |
| Anticipated shame |  |  |  |  |  |  |  |  |  |  |  |
| Low/med (score < 3.75) | 76 | 47 | 36 | 42 | 1 | 39 | 43 | 1 | 47 | 47 | 1 |
| High (score ≥ 3.75) | 87 | 53 | 50 | 58 | 1.54 (0.83-2.89) | 52 | 57 | 1.45 (0.78-2.71) | 54 | 54 | 1.03 (0.55-1.95) |
| Impulsiveness |  |  |  |  |  |  |  |  |  |  |  |
| Low/med (score < 2.63) | 78 | 48 | 39 | 45 | 1 | 35 | 39 | 1 | 45 | 45 | 1 |
| High (score ≥ 2.63) | 85 | 52 | 47 | 55 | 1.27 (0.68-2.36) | 56 | 62 | **2.44 (1.30-4.65)** | 56 | 55 | 1.45 (0.77-2.75) |
| Social norms and support |  |  |  |  |  |  |  |  |  |  |  |
| Low/med (score < 3.20) | 69 | 42 | 34 | 40 | 1 | 35 | 39 | 1 | 44 | 44 | 1 |
| High (score ≥ 3.20) | 94 | 58 | 52 | 61 | 1.25 (0.67-2.34) | 56 | 62 | 1.41 (0.75-2.64) | 57 | 56 | 0.86 (0.45-1.63) |
| Knowledge^b^ |  |  |  |  |  |  |  |  |  |  |  |
| Low/med (score < 6.00) | 91 | 56 | 48 | 56 | 1 | 56 | 62 | 1 | 60 | 59 | 1 |
| High (score ≥ 6.00) | 72 | 44 | 38 | 44 | 0.97 (0.52-1.82) | 35 | 39 | *0.57 (0.30-1.07)* | 41 | 41 | 0.67 (0.35-1.26) |
| Self-efficacy |  |  |  |  |  |  |  |  |  |  |  |
| Low/med (score < 3.00) | 64 | 39 | 36 | 42 | 1 | 32 | 35 | 1 | 40 | 40 | 1 |
| High (score ≥ 3.00) | 99 | 61 | 50 | 58 | 0.78 (0.41-1.46) | 59 | 65 | 1.45 (0.77-2.74) | 61 | 60 | 0.95 (0.49-1.81) |
| Self-esteem |  |  |  |  |  |  |  |  |  |  |  |
| Low/med (score < 3.74) | 54 | 33 | 27 | 31 | 1 | 28 | 31 | 1 | 35 | 35 | 1 |
| High (score ≥ 3.74) | 109 | 67 | 59 | 69 | 1.23 (0.63-2.37) | 63 | 69 | 1.32 (0.68-2.56) | 66 | 65 | 0.86 (0.43-1.68) |
| Risk perception for CT (own risk) |  |  |  |  |  |  |  |  |  |  |  |
| Low/med (score < 27.50) | 44 | 51 | 84 | 52 | 1 | 52 | 57 | 1 | 58 | 57 | 1 |
| High (score ≥ 27.50) | 42 | 49 | 79 | 48 | 1.01 (0.54-1.87) | 39 | 43 | *0.58 (0.31-1.09)* | 43 | 43 | **0.52 (0.27-0.99)** |
| CT infection |  |  |  |  |  |  |  |  |  |  |  |
| No | 134 | 82 | 71 | 83 | 1 | 73 | 80 | 1 | 83 | 82 | 1 |
| Yes | 29 | 18 | 15 | 17 | 0.89 (0.39-2.02) | 18 | 20 | 1.29 (0.57-2.04) | 18 | 18 | 0.95 (0.42-2.24) |

* P-value < 0.1, but number of observations in each cell too small to include in multivariable model

^a^ Attitudes regarding prevention of chlamydia

^b^ Knowledge regarding sexual health, prevention of chlamydia and consequences of chlamydia diagnosis

Footnote: Categories do not all add up to 100%, as missing values are not shown. Statistical associations are shown in in italic when the p-value is equal to or smaller than 0.1, and in bold when the p-value is equal to or smaller than 0.05.

Abbreviations: OR = crude odds ratio, CI = Confidence Interval; Low/med = Low/medium, CT = Chlamydia; STI = Sexually Transmitted Infection.
