# Supplementary material for: Who drops out and when? Predictors of non-response and loss to follow-up in a longitudinal cohort study among STI clinic visitors
Source: PLoS One. 2019 Jun 19;14(6):e0218658. doi: 10.1371/journal.pone.0218658 (PMC6583983; doi:10.1371/journal.pone.0218658)
Supplement: S3 Table — (DOCX) [file pone.0218658.s003.docx]

**S3 Table. Multivariable logistic regression analyses of demographic and sexual health-related predictors of non-response at the three follow-up data collection moments**

|  | *Baseline* | | *3-week follow-up non-response* | | | *6-month follow-up non-response* | | | *1-year follow-up non-response* | | |
| --- | --- | --- | --- | --- | --- | --- | --- | --- | --- | --- | --- |
|  | N | % | N | % | *aOR* | N | % | *aOR* | N | % | *aOR* |
|  |  |  |  |  | *(95%CI)* |  |  | *(95%CI)* |  |  | *(95%CI)* |
| Total | 810 |  | 378 | 47 |  | 394 | 49 |  | 466 | 57 |  |
| Age |  |  |  |  |  |  |  |  |  |  |  |
| 18-20 years | 179 | 22 | 86 | 23 | - | 99 | 25 | 1 | 108 | 23 | - |
| 21-22 years | 297 | 37 | 143 | 38 | - | 153 | 39 | 0.89 (0.61-1.30) | 175 | 38 | - |
| 23-24 years | 334 | 41 | 149 | 39 | - | 142 | 36 | **0.62 (0.42-0.89)** | 183 | 39 | - |
| Gender |  |  |  |  |  |  |  |  |  |  |  |
| Female | 651 | 80 | 284 | 75 | 1 | 293 | 74 | 1 | 348 | 75 | 1 |
| Male | 159 | 20 | 94 | 25 | **1.86 (1.31-2.65)** | 101 | 26 | **2.14 (1.49-3.10)** | 118 | 25 | **2.51 (1.71-3.74)** |
| Education level |  |  |  |  |  |  |  |  |  |  |  |
| Low/medium | 84 | 10 | 48 | 13 | 1 | 53 | 14 | 1 | 61 | 13 | 1 |
| High | 725 | 90 | 330 | 87 | *0.65 (0.41-1.03)* | 340 | 86 | **0.59 (0.36-0.94)** | 405 | 87 | **0.49 (0.29-0.81)** |
| Migration background |  |  |  |  |  |  |  |  |  |  |  |
| Dutch | 656 | 81 | 303 | 80 | - | 309 | 78 | 1 | 367 | 79 | 1 |
| Non-Dutch | 154 | 19 | 75 | 20 | - | 85 | 22 | *1.42 (0.99-2.04)* | 99 | 21 | *1.43 (0.99-2.08)* |
| Symptoms |  |  |  |  |  |  |  |  |  |  |  |
| No | 684 | 84 | 319 | 84 | - | 321 | 82 | 1 | 397 | 85 | - |
| Yes | 126 | 16 | 59 | 16 | - | 73 | 18 | *1.45 (0.98-2.17)* | 69 | 15 | - |

Footnote: Categories do not all add up to 100%, as missing values are not shown. Statistical associations are shown in in italic when the p-value is equal to or smaller than 0.1, and in bold when the p-value is equal to or smaller than 0.05. Only variables that were pre-selected in the univariable analyses are shown here.

Abbreviations: aOR = adjusted odds ratio, CI = Confidence Interval.
