# Supplementary material for: Who drops out and when? Predictors of non-response and loss to follow-up in a longitudinal cohort study among STI clinic visitors
Source: PLoS One. 2019 Jun 19;14(6):e0218658. doi: 10.1371/journal.pone.0218658 (PMC6583983; doi:10.1371/journal.pone.0218658)
Supplement: S1 File — (PDF) [file pone.0218658.s004.pdf]

Rijksinstituut voor Volksgezondheid
en Milieu
Ministerie van Volksgezondheid,
Welzijn en Sport

De impact van denken en doen op chlamydia verspreiding (iMPaCT)

Wij willen weten hoe jij denkt over seks, gezondheid en soa-testen. Het invullen van de online vragenlijst duurt ongeveer 10 minuten en je helpt ons hiermee de zorg rond seksualiteit voor jongeren te verbeteren! Je informatie wordt vertrouwelijk behandeld en niet doorgegeven aan anderen. Wat levert meedoen jou op? Je krijgt een gratis zelfafnametest na 6 maanden en als je mee doet aan het hele onderzoek krijg je een bol.com cadeaukaart.

Mocht je vragen of eventuele problemen hebben bij het invullen van de vragenlijst? Neem dan contact op met de onderzoekers van iMPaCT via e-mail impact@rivm.nl of via telefoonnummer 030 - 274 2538 (alleen tijdens kantooruren).

iMPaCT vragenlijst

Afkortingen en definities van gebruikte termen in de vragenlijst

Om ervoor te zorgen dat iedereen weet wat de termen die in de vragenlijst gebruikt worden betekenen, worden deze hieronder kort toegelicht. Lees deze uitleg alsjeblieft goed. Als je alles hebt gelezen kun je beginnen met de vragenlijst.

Soa: Seksueel overdraagbare aandoeningen (geslachtsziekten).
Vaginale seks: De penis van een man in de vagina van een vrouw.
Anale seks: De penis van een man in de anus (rectum) van de vrouw.
Orale seks: De mond van een man/vrouw op de geslachtsorganen van zijn/haar partner.
Seks: Alléén vaginale en/of anale seks.
Partner(s): Mannen/vrouwen waar je eenmalig of vaker seks mee hebt gehad.

1. In hoeverre ben je het eens met de volgende stellingen?

	Helemaal mee oneens				Helemaal mee eens
Ik ben gemotiveerd om gezond te blijven.	1	2	3	4	5
Het is voor mij belangrijk om tijdens de seks een condoom te gebruiken.	1	2	3	4	5
Ik ben minder dan gemiddeld met mijn gezondheid bezig.	1	2	3	4	5
Mijn gezondheid is belangrijk voor me.	1	2	3	4	5
Ik ben gemotiveerd om mezelf tijdens de seks te beschermen.	1	2	3	4	5
Ik vind het belangrijk te voorkomen dat ik een soa krijg.	1	2	3	4	5

2. Hoe denk je over de volgende stelling?

"Ik vind voorkómen om chlamydia te krijgen..."

Heel onbelangrijk	1	2	3	4	5	Heel belangrijk
Heel overbodig	1	2	3	4	5	Heel noodzakelijk
Heel nutteloos	1	2	3	4	5	Heel nuttig
Heel onwenselijk	1	2	3	4	5	Heel wenselijk

3. In hoeverre ben je het eens met de volgende stellingen?

	Helemaal mee oneens				Helemaal mee eens
Ik verwacht dat ik in de toekomst altijd een condoom zal gebruiken.	1	2	3	4	5
Ik verwacht dat ik in de toekomst geen seks zal hebben wanneer ik geen condoom bij de hand heb.	1	2	3	4	5
Mijn toekomstige partner(s) moet(en) getest zijn op soa voor we seks hebben zonder condoom.	1	2	3	4	5

4. Voor het beantwoorden van de onderstaande vragen, vragen we je om een schatting te geven van de kans op bepaalde gebeurtenissen, als een percentage tussen 0% en 100%.

Hoe groot is de kans om chlamydia te krijgen als je één keer seks met iemand hebt zonder condoom?		%
Hoe groot is de kans om hiv te krijgen als je één keer seks met iemand hebt zonder condoom?		%
Hoe groot is jouw kans om komend jaar chlamydia te krijgen?		%
Hoe groot is jouw kans om ooit chlamydia te krijgen?		%
Hoe groot is jouw kans om komend jaar hiv te krijgen?		%
Hoe groot is jouw kans om ooit hiv te krijgen?		%

5. Voor het beantwoorden van de onderstaande vragen, vragen we je om een schatting te geven van de kans op bepaalde gebeurtenissen, als een percentage tussen 0% en 100%. Met een gemiddelde leeftijdsgenoot bedoelen we mensen in jouw sociale omgeving, zoals vrienden die op jou lijken.

Hoe groot is de kans dat een gemiddelde leeftijdsgenoot in het komende jaar chlamydia krijgt?		%
Hoe groot is de kans dat een gemiddelde leeftijdsgenoot ooit chlamydia krijgt?		%
Hoe groot is de kans dat een gemiddelde leeftijdsgenoot in het komende jaar hiv krijgt?		%
Hoe groot is de kans dat een gemiddelde leeftijdsgenoot ooit hiv krijgt?		%

6. Zijn de volgende stellingen 'waar' of 'niet waar'? Als je het antwoord niet weet, omcirkel dan de optie 'weet ik niet'.

De anticonceptiepil verkleint de kans op chlamydia.	Waar	Niet waar	Weet ik niet
Als je je na seks goed wast, loop je minder snel chlamydia op.	Waar	Niet waar	Weet ik niet
Als je geen (lichamelijke) klachten hebt, kun je toch chlamydia hebben.	Waar	Niet waar	Weet ik niet
Chlamydia kan onvruchtbaarheid bij vrouwen veroorzaken.	Waar	Niet waar	Weet ik niet
Je kan geen chlamydia krijgen van één keer seks zonder condoom.	Waar	Niet waar	Weet ik niet
Je kan chlamydia krijgen van anale seks zonder condoom.	Waar	Niet waar	Weet ik niet

7. In hoeverre ben je het eens met de volgende stellingen?

"Als ik chlamydia heb, zouden mensen..."

	Helemaal mee oneens				Helemaal mee eens
mij vermijden.	1	2	3	4	5
slecht over mij denken.	1	2	3	4	5
denken dat ik vies ben.	1	2	3	4	5
geen vrienden met me willen zijn.	1	2	3	4	5
mij afwijzen.	1	2	3	4	5
om me heen zich ongemakkelijk voelen.	1	2	3	4	5

8. In hoeverre ben je het eens met de volgende stellingen?

"Als ik chlamydia heb, zou ik ..."

	Helemaal mee oneens				Helemaal mee eens
me schamen.	1	2	3	4	5
me generen.	1	2	3	4	5
me schuldig voelen.	1	2	3	4	5
bang zijn.	1	2	3	4	5
balen van mezelf.	1	2	3	4	5

9. In hoeverre zijn deze stellingen op jou van toepassing?

	Helemaal mee oneens				Helemaal mee eens
Als ik me ongelukkig voel, doe ik vaak dingen zonder na te denken.	1	2	3	4	5
Als ik me afgewezen voel, zeg ik vaak dingen waar ik later spijt van krijg.	1	2	3	4	5
Vaak maak ik dingen erger als ik me ongelukkig voel, omdat ik handel zonder na te denken.	1	2	3	4	5
In een opwelling zeg ik vaak dingen waar ik later spijt van krijg.	1	2	3	4	5

10. In hoeverre zijn deze stellingen op jou van toepassing?

	Helemaal mee oneens				Helemaal mee eens
Ik houd er van om activiteiten volledig af te ronden.	1	2	3	4	5
Ik maak af waar ik aan begonnen ben.	1	2	3	4	5
Ik ben iemand die hard werkt om dingen af te krijgen.	1	2	3	4	5
Als ik een project begonnen ben, krijg ik het bijna altijd af.	1	2	3	4	5

11. In hoeverre zijn deze stellingen op jou van toepassing?

	Helemaal mee oneens				Helemaal mee eens
Ik denk meestal zorgvuldig en doelgericht na.	1	2	3	4	5
Voordat ik iets doe denk ik meestal zorgvuldig na.	1	2	3	4	5
Ik neem mijn beslissingen na zorgvuldig nadenken.	1	2	3	4	5
Voordat ik een besluit neem, overweeg ik eerst alle voor- en nadelen.	1	2	3	4	5

12. In hoeverre zijn deze stellingen op jou van toepassing?

	Helemaal mee oneens				Helemaal mee eens
Ik vind het heerlijk om risico's te nemen.	1	2	3	4	5
Ik ben altijd in voor nieuwe en opwindende ervaringen, ook al zijn ze een beetje gevaarlijk of ongebruikelijk.	1	2	3	4	5
Ik houd er soms van dingen te doen die een beetje eng zijn.	1	2	3	4	5
Ik zoek nieuwe en opwindende ervaringen op.	1	2	3	4	5

13. In hoeverre ben je het eens met de volgende stellingen?

	Helemaal mee oneens				Helemaal mee eens
Ik vind het moeilijk om de seks te onderbreken om een condoom om te doen.	1	2	3	4	5
Ik vind het moeilijk om te onthouden een condoom bij me te hebben.	1	2	3	4	5
Ik vind het vervelend om condooms te kopen.	1	2	3	4	5
Als ik onder invloed ben van alcohol of drugs, vergeet ik snel een condoom te gebruiken.	1	2	3	4	5

14. In hoeverre ben je het eens met de volgende stellingen?

Met ‘vrienden’ bedoelen we mensen waar je geen seks mee hebt.

	Helemaal mee oneens				Helemaal mee eens
Mijn vrienden vinden dat ik condooms moet gebruiken.	1	2	3	4	5
Mijn meest recente partner vindt dat ik condooms moet gebruiken.	1	2	3	4	5
Mijn vrienden doen eerst een soa-test voordat ze seks met iemand hebben zonder condoom.	1	2	3	4	5
Als ik chlamydia blijk te hebben, zullen mijn vrienden me steunen.	1	2	3	4	5
Als ik chlamydia blijk te hebben, zal mijn meest recente partner mij steunen.	1	2	3	4	5

15. In hoeverre ben je het eens met de volgende stellingen?

	Helemaal mee oneens				Helemaal mee eens
Ik heb veel zelfvertrouwen	1	2	3	4	5

16. Hoe oud was je toen je voor het eerst seks had?

Hiermee bedoelen we de eerste partner waar je seks mee had vanaf je 13e.

☐ jaar oud

17. Hoeveel verschillende partners heb je je hele leven gehad?

Hiermee bedoelen we je vaste partner(s) en losse contacten waarmee je seks hebt gehad. Geef een schatting als je het niet meer precies weet.

☐ partner(s)

18. Hoeveel verschillende partners heb je in het afgelopen jaar gehad?

Hiermee bedoelen we je vaste partner(s) en losse contacten waarmee je seks hebt gehad. Geef een schatting als je het niet meer precies weet.

☐ partner(s)

19. Hoeveel verschillende partners heb je in de afgelopen 6 maanden gehad?

Hiermee bedoelen we je vaste partner(s) en losse contacten waarmee je seks hebt gehad. Geef een schatting als je het niet meer precies weet.

☐ partner(s)

Wanneer je geen partner hebt gehad in de afgelopen 6 maanden, ga verder naar vraag 21.

20. Met hoeveel van deze partners had je in de afgelopen 6 maanden voor het eerst seks?

Hiermee bedoelen we dat je met deze partner vóór het afgelopen half jaar nog niet eerder seks hebt gehad. Geef een schatting als je het niet meer precies weet.

☐ partner(s)

21. Hoe vaak gebruik je condooms tijdens seks?

- ☐ Nooit
- ☐ Meestal niet
- ☐ Soms wel, soms niet
- ☐ Meestal wel
- ☐ Altijd

Wanneer je geen partner hebt gehad in de afgelopen 6 maanden, ga verder naar vraag 24.

22. Hoe vaak heb je in de afgelopen 4 weken seks gehad?

Geef een schatting als je het niet meer precies weet.

☐ keer

Wanneer je niet meer dan 1 partner hebt gehad in de afgelopen 6 maanden, ga verder naar vraag 24.

23. Hoeveel verschillende partners heb je in de afgelopen 4 weken gehad?

☐ partner(s)

De volgende vragen zullen gaan over de partner(s) waar je het meest recent seks hebt gehad. Dit kan iemand zijn waar je één keer of een paar keer seks mee hebt gehad, maar ook een vaste partner.

24. Partner 1

Denk aan de partner waar je het meest recent seks mee hebt gehad. Je kunt bij de onderstaande vragen de initialen of een bijnaam gebruiken van de partner. Niemand kan deze initialen of bijnamen zien behalve jij.

Initialen of bijnaam ☐

25. Wanneer heb je voor het laatst seks gehad met Partner1?

Geef een schatting als je het niet meer precies weet.

☐ dd/mm/jjjj

26. Hebben jij en Partner1 toen een condoom gebruikt?

- ☐ Ja
- ☐ Nee
- ☐ Weet ik niet

27. Welke van de volgende omschrijvingen past het beste bij jou en Partner1 toen jullie voor het laatst seks hadden?

- ☐ Getrouwd/geregistreerd partnerschap
- ☐ Vaste relatie, samenwonend
- ☐ Vaste relatie, niet samenwonend
- ☐ Ex-partner
- ☐ Kennissen/vrienden
- ☐ Recent ontmoet
- ☐ Eerste ontmoeting
- ☐ Anders, namelijk __________

28. Is het waarschijnlijk dat je in de toekomst nog seks hebt met Partner1?

- ☐ Ja
- ☐ Waarschijnlijk wel
- ☐ Waarschijnlijk niet
- ☐ Nee
- ☐ Weet ik niet

29. Was de laatste keer dat je seks had met Partner1 ook de eerste keer?

- ☐ Ja, we hebben eenmalig seks met elkaar gehad — *Ga verder naar vraag 32*
- ☐ Nee, we hebben vaker seks met elkaar gehad
- ☐ Weet ik niet — *Ga verder naar vraag 32*

30. Wanneer had je voor het eerst seks met Partner1?

Geef een schatting als je het niet meer precies weet.

________ dd/mm/jjjj

31. Hoe vaak heb je in de afgelopen 4 weken seks gehad met Partner1?

Geef een schatting als je het niet meer precies weet.

______ keer

32. Wat voor seks hebben jij en Partner1 in de afgelopen 4 weken gehad?

Meerdere antwoorden mogelijk.

- ☐ Vaginale seks
- ☐ Anale seks
- ☐ Orale seks
- ☐ Niet van toepassing

33. Wat is de leeftijd van Partner1?

Geef een schatting als je het niet meer precies weet.

[ ] jaar oud

34. In welk land/gebied is Partner1 geboren?

- ☐ Nederland
- ☐ Turkije
- ☐ Marokko
- ☐ Suriname
- ☐ Nederlandse Antillen
- ☐ Aruba
- ☐ Afrika
- ☐ Zuid-Amerika
- ☐ Midden-Amerika
- ☐ Oost-Europa
- ☐ Azië
- ☐ Weet ik niet
- ☐ Een ander land, namelijk [ ]

35. Denk je dat Partner1 seks met iemand anders heeft gehad in de periode waarin hij/zij seks had met jou?

- ☐ Ja
- ☐ Waarschijnlijk wel
- ☐ Waarschijnlijk niet
- ☐ Nee
- ☐ Weet ik niet

36. Partner 2

Denk nu aan de partner waar je vóór Partner1 het meest recent seks mee hebt gehad. Je kunt bij de volgende vragen weer de initialen of een bijnaam gebruiken van de partner.

Initialen of bijnaam [ ]

37. Wanneer heb je voor het laatst seks gehad met Partner2?

Geef een schatting als je het niet meer precies weet.

[ ] dd/mm/jjjj

38. Was de laatste keer dat je seks had met Partner2 ook de eerste keer?

- ☐ Ja, we hebben eenmalig seks met elkaar gehad — *Ga verder naar vraag 41*
- ☐ Nee, we hebben vaker seks met elkaar gehad — *Sla vraag 41 over*
- ☐ Weet ik niet — *Sla vraag 41 over*

39. Wanneer had je voor het eerst seks met Partner2?

Geef een schatting als je het niet meer precies weet.

[ ] dd/mm/jjjj

40. Hoe vaak gebruikten jij en Partner2 condooms?

- ☐ Nooit
- ☐ Meestal niet
- ☐ Soms wel, soms niet
- ☐ Meestal wel
- ☐ Altijd

41. Hebben jij en Partner2 toen een condoom gebruikt?

- ☐ Ja
- ☐ Nee
- ☐ Weet ik niet

42. Hoe vaak heb je in de afgelopen 4 weken seks gehad met Partner2?

Geef een schatting als je het niet meer precies weet.

[ ] keer

Bedankt voor het invullen van de vragenlijst!
