# Supplementary material for: Who drops out and when? Predictors of non-response and loss to follow-up in a longitudinal cohort study among STI clinic visitors
Source: PLoS One. 2019 Jun 19;14(6):e0218658. doi: 10.1371/journal.pone.0218658 (PMC6583983; doi:10.1371/journal.pone.0218658)
Supplement: S2 File — (PDF) [file pone.0218658.s005.pdf]

Rijksinstituut voor Volksgezondheid
en Milieu
*Ministerie van Volksgezondheid,
Welzijn en Sport*

The impact of thinking and doing on the spread of chlamydia (iMPaCT)

We would like to know how you think about sex, health, and STI testing. Filling out the online questionnaire will take about 10 minutes and you help us improve (sexual) health care for adolescents! Your personal information will be treated confidentially and will not be passed on to others. What are the benefits of participating for you? You will receive a free self-sampling test kit after 6 months and if you participate in the entire study you will receive a gift card.

If you have any questions or encounter problems while filling out the questionnaire? Contact the researchers of the iMPaCT study via e-mail impact@rivm.nl or via telephone 030 - 274 2538 (only during office hours).

iMPaCT questionnaire

Abbreviations and definitions of terms used in the questionnaire

So that everyone attaches the same meaning to these terms, they are explained below.

Pleas be sure to read these explanations.

STI: Sexually Transmitted Infection.
Vaginal sex: A man's penis in a woman's vagina.
Anal sex: A man's penis in a woman's anus (rectum or back passage).
Oral sex: A woman/man's or a man/woman's mouth on a partner's genital area.
Sex: This includes vaginal and/or anal sex
Partner(s): People who have had sex together, whether just once, or a few times, or as steady partner(s).

1. To what extent do you agree with the following statements?

	Strongly disagree				Strongly agree
I am motivated to stay healthy.	1	2	3	4	5
It is important to me to use a condom when having sex.	1	2	3	4	5
I worry less than average about my health.	1	2	3	4	5
My health is important to me.	1	2	3	4	5
I am motivated to protect myself during sex.	1	2	3	4	5
It is important to me to prevent myself from contracting an STI.	1	2	3	4	5

2. To what extent do you agree with the following statements?

"I think prevention of chlamydia is…"

Very unimportant	1	2	3	4	5	Very important
Very unnecessary	1	2	3	4	5	Very necessary
Very useless	1	2	3	4	5	Very useful
Very undesirable	1	2	3	4	5	Very desirable

3. To what extent do you agree with the following statements?

	Strongly disagree				Strongly agree
I expect that I will always use a condom in the future.	1	2	3	4	5
I expect I will not have sex in the future, when I do not have a condom with me.	1	2	3	4	5
My future partner(s) need to take an STI test before we have condomless sex.	1	2	3	4	5

4. To answer the questions below, we ask you to give an estimate of the probability of certain events, as a percentage between 0% and 100%.

What is the probability of contracting chlamydia from a single condomless sex act?		%
What is the probability of contracting HIV from a single condomless sex act?		%
How likely do you think it is that you will get chlamydia in the coming year?		%
How likely do you think it is that you will get chlamydia in your lifetime?		%
How likely do you think it is that you will get HIV in the coming year?		%
How likely do you think it is that you will get HIV in your lifetime?		%

5. To answer the questions below, we ask you to give an estimate of the probability of certain events, as a percentage between 0% and 100%. With peers we mean people in your social environment, such as your friends.

How likely do you think it is that your peers will get chlamydia in the coming year?		%
How likely do you think it is that your peers will get chlamydia in their lifetime?		%
How likely do you think it is that your peers will get HIV in the coming year?		%
How likely do you think it is that your peers will get HIV in their lifetime?		%

6. Are the following statements 'true' of 'false'? If you don't know the answer, circle the option 'I don't know'.

The anti-conception pill decreases a person's risk of getting chlamydia.	True	False	I don't know
You can prevent an STI by washing well after sex.	True	False	I don't know
Even if you don't have any (physical) symptoms, you can still have chlamydia.	True	False	I don't know
Chlamydia can lead to infertility in women.	True	False	I don't know
You can't get chlamydia from a single condomless sex act.	True	False	I don't know
You can get chlamydia during condomless anal sex.	True	False	I don't know

7. To what extent do you agree with the following statements?

"If I have chlamydia, people would..."

	Strongly disagree				Strongly agree
avoid me.	1	2	3	4	5
think I am weak and foolish.	1	2	3	4	5
think I am unclean.	1	2	3	4	5
not want to be friends with me.	1	2	3	4	5
reject me.	1	2	3	4	5
be uncomfortable around me.	1	2	3	4	5

8. To what extent do you agree with the following statements?

"If I have chlamydia, I would..."

	Strongly disagree				Strongly agree
be ashamed.	1	2	3	4	5
be embarrassed.	1	2	3	4	5
feel guilty.	1	2	3	4	5
be scared.	1	2	3	4	5
be disappointed in myself.	1	2	3	4	5

9. To what extent do you agree with the following statements?

	Strongly disagree				Strongly agree
When I am upset, I often act without thinking.	1	2	3	4	5
When I feel rejected, I will often say things that I later regret.	1	2	3	4	5
Sometimes when I feel bad, I can't seem to stop what I am doing even though it is making me feel worse.	1	2	3	4	5
I blurt out things without thinking.	1	2	3	4	5

10. To what extent do you agree with the following statements?

	Strongly disagree				Strongly agree
Once I get going on something I hate to stop.	1	2	3	4	5
I finish what I start.	1	2	3	4	5
I pace myself to get things done on time.	1	2	3	4	5
I almost always finish projects I start.	1	2	3	4	5

11. To what extent do you agree with the following statements?

	Strongly disagree				Strongly agree
My thinking is usually careful and purposeful.	1	2	3	4	5
I usually think carefully before doing anything.	1	2	3	4	5
I like to stop and think things over before I do them.	1	2	3	4	5
I usually make up my mind after considering all advantages and disadvantages.	1	2	3	4	5

12. To what extent do you agree with the following statements?

	Strongly disagree				Strongly agree
I quite enjoy taking risks.	1	2	3	4	5
I welcome new and exciting experiences and sensations, even if they are a little frightening and unconventional.	1	2	3	4	5
I like doing things that are a bit frightening.	1	2	3	4	5
I seek new and exciting experiences.	1	2	3	4	5

13. To what extent do you agree with the following statements?

	Strongly disagree				Strongly agree
I do not feel confident in my ability to interrupt sex to put on a condom.	1	2	3	4	5
I do not feel confident I could remember to carry a a condom with me should I need one.	1	2	3	4	5
I do not feel confident I could purchase condoms without feeling embarrassed.	1	2	3	4	5
If I am under the influence of alcohol or drugs, I often forget to use a condom.	1	2	3	4	5

14. To what extent do you agree with the following statements?

With 'friends' we mean people you don't have sex with.

	Strongly disagree				Strongly agree
My friends think that I should use condoms.	1	2	3	4	5
My most recent partner thinks that I should use condoms.	1	2	3	4	5
My friends take an STI test before they have unprotected sex with someone.	1	2	3	4	5
If I were to be diagnosed with chlamydia, my friends will support me.	1	2	3	4	5
If I were to be diagnosed with chlamydia, my most recent partner will support me.	1	2	3	4	5

15. To what extent do you agree with the following statements?

	Strongly disagree				Strongly agree
I have high self-esteem.	1	2	3	4	5

16. How old were you when you first had sex with someone?

By this we mean the first partner that you had sex with since you were 13 years old.

☐ years old

17. Altogether, in your life so far, how many partners have you had sex with?

By this we mean your steady partner(s) and casual partner(s) with whom you have had sex. If you are not sure of the exact number please give your best estimate.

☐ partner(s)

18. Altogether, in the past year, how many partners have you had sex with?

By this we mean your steady partner(s) and casual partner(s) with whom you have had sex. If you are not sure of the exact number please give your best estimate.

☐ partner(s)

19. Altogether, in the past six months, how many partners have you had sex with?

By this we mean your steady partner(s) and casual partner(s) with whom you have had sex. If you are not sure of the exact number please give your best estimate.

☐ partner(s)

If you have not had sex in the past six months, go to question 21.

20. How many of these partners were *new* partners who you had sex with for the first time during the past six months?

If you are not sure of the exact number please give your best estimate.

☐ partner(s)

21. How often do you use condoms during sex?

- ☐ Never
- ☐ Not very often
- ☐ Sometimes
- ☐ Very often
- ☐ Always

If you have not had sex in the past six months, go to question 24.

22. How often have you had sex in the past four weeks?

If you are not sure of the exact number please give your best estimate.

☐ time(s)

If you have not had more than one partner in the past six months, go to question 24.

23. Altogether, in the past four weeks, how many partners have you had sex with?

☐ partner(s)

The following questions will be about the partner(s) with whom you have had sex most recently, whether it was just once, a few times, or with a steady partner.

24. Partner 1

Think about your most recent partner. To make it easier to remember the answers to these questions, please type in a nickname or an initial for the person you had sex with most recently. This is just to help you remember who you are answering the questions about. No one will see this nickname or initial except you.

Nickname or initials ☐

25. When was the most recent occasion you had sex with Partner 1?

If not sure of the exact month or year please give your best estimate.

☐ dd/mm/yyyy

26. Did you and Partner 1 use a condom on that most recent occasion?

☐ Yes
☐ No
☐ I don't know

27. Which one of these descriptions applies best to you and Partner 1 at the time you most recently had sex?

☐ Married/registered partnership
☐ Steady partner, living together
☐ Steady partner, not living together
☐ Ex-partner
☐ Acquaintance/friends
☐ We had recently met
☐ We had just met for the first time
☐ Other, ... [ ]

28. Are you likely to have sex with Partner 1 again in the future?

☐ Yes
☐ Probably
☐ Probably not
☐ No
☐ I don't know

29. Was the most recent occasion you had sex with Partner 1 also the first occasion with (him/her) or not?

☐ Yes, I have only had sex with (him/her) once *Go to question 32*
☐ No, I have had with (him/her) on more than one occasion
☐ I don't know *Go to question 32*

30. When was the first occasion with Partner 1?

If not sure of the exact month or year please give your best estimate.

[ ] dd/mm/yyyy

31. How often have you had sex with Partner 1 in the past four weeks?

If you are not sure of the exact number please give your best estimate.

[ ] time(s)

32. What type(s) of sex have you had with Partner 1 in the past four weeks?

Multiple answers possible.

- ☐ Vaginal sex
- ☐ Anal sex
- ☐ Oral sex
- ☐ Not applicable

33. How old was Partner 1 when you had sex?

If you are not sure of the exact age please give your best estimate.

[ ] years old

34. In which country/region was Partner 1 born?

- ☐ Netherlands
- ☐ Turkey
- ☐ Morocco
- ☐ Suriname
- ☐ Netherlands Antilles
- ☐ Aruba
- ☐ Africa
- ☐ South-America
- ☐ Central-America
- ☐ Eastern-Europe
- ☐ Asia
- ☐ I don't know
- ☐ Another country, ... [ ]

35. Do you think Partner 1 had sex with anyone else in the time between when you first and most recently had sex together?

- ☐ Yes
- ☐ Probably
- ☐ Probably not
- ☐ No
- ☐ I don't know

36. Partner 2

Think about your second most recent partner. To make it easier to remember the answers to these questions, please type in a nickname or an initial again, now for your second most recent partner.

Nickname or initials [ ]

37. When was the most recent occasion you had sex with Partner 2?

If not sure of the exact month or year please give your best estimate.

[ ] dd/mm/yyyy

38. Was the most recent occasion you had sex with Partner 2 also the first occasion with (him/her) or not?

- ☐ Yes, I have only had sex with (him/her) once — *Go to question 41*
- ☐ No, I have had sex with (him/her) on more than one occasion — *Skip question 41*
- ☐ I don't know — *Skip question 41*

39. When was the first occasion with Partner2?

If not sure of the exact month or year please give your best estimate.

[ ] dd/mm/yyyy

40. How often have you and Partner 2 used condoms during sex?

- ☐ Never
- ☐ Not very often
- ☐ Sometimes
- ☐ Very often
- ☐ Always

41. Did you and Partner 2 use a condom?

- ☐ Yes
- ☐ No
- ☐ I don't know

42. How often have you had sex with Partner 2 in the past four weeks?

If not sure of the exact number please give your best estimate.

[ ] time(s)

Thank you for filling out the questionnaire!
